# Immobilization of *Trichoderma harzianum* α-Amylase on Treated Wool: Optimization and Characterization

**DOI:** 10.3390/molecules19068027

**Published:** 2014-06-13

**Authors:** Saleh A. Mohamed, Jalaluddin A. Khan, Omar A. M. Al-Bar, Reda M. El-Shishtawy

**Affiliations:** 1 Biochemistry Department, Faculty of Science, King Abdulaziz University, 21589, Jeddah, Kingdom of Saudi Arabia; E-Mails: jalalawlia@yahoo.com (J.A.K.); al_baromar@hotmail.com (O.A.M.A.-B.); 2 Molecular Biology Department, National Research Center, Dokki, 12622, Cairo, Egypt; 3 Chemistry Department, Faculty of Science, King Abdulaziz University, 21589, Jeddah, Kingdom of Saudi Arabia; E-Mail: elshishtawy@hotmail.com; 4 Dyeing, Printing and Textile Auxiliaries Department, Textile Research Division, National Research Center, Dokki, 12622, Cairo, Egypt

**Keywords:** *Trichoderma harzianum*, α-amylase, immobilized enzyme, optimization

## Abstract

α-Amylase from *Trichoderma harzianum* was covalently immobilized on activated wool by cyanuric chloride. Immobilized α-amylase exhibited 75% of its initial activity after 10 runs. The soluble and immobilized α-amylases exhibited maximum activity at pH values 6.0 and 6.5, respectively. The immobilized enzyme was more thermally stable than the soluble one. Various substrates were hydrolyzed by immobilized α-amylase with high efficiencies compared to those of soluble α-amylase. The inhibition of the immobilized α-amylase by metal ions was low as compared with soluble enzyme. On the basis of the results obtained, immobilized α-amylase could be employed in the saccharification of starch processing.

## 1. Introduction

α-Amylase (EC 3.2.1.1; α-1,4-d-glucan glucanohydrolase) hydrolyzes α-1,4-glucosidic bonds in starch, amylopectin, and glycogen in an *endo* fashion and forms low molecular weight products. Starch hydrolyzates with high-dextrose content are extensively used in the food industry and as a source of fermentable sugars. The industrial preparation of glucose syrups involves a preliminary starch saccharification to maltodextrin using α-amylase, followed by a second hydrolysis to glucose using glucoamylase. α-Amylases are applied in several biotechnological applications such as food processing, textile, paper and pharmaceutical industries. [1]. Microbial amylases have almost completely replaced chemical hydrolysis of starch in the starch processing industry [2]. In fact amylolytic enzymes account for almost 25% of the global enzyme sales [3,4].

Immobilized enzymes have several advantages over soluble enzymes. Immobilization of enzymes minimizes the inhibition by substrates, reaction products, inhibitors, solvents, detergents or any environmental conditions. In addition, enzyme immobilization results in good operational and storage stability, high sensitivity, high selectivity, short response times and high reproducibility [5,6]. Like many other enzymes, immobilized amylase would gain improved stability and reusability [7,8]. Multipoint and multisubunit covalent immobilization improve the stability of monomeric or multimeric enzymes [9,10]. Various nanomaterials and nanostructures generally provide a large surface area and low mass-transfer resistance, which enables better interaction with the enzyme, increases immobilization efficiency, and enhances the long-term storage and recycling stability of the enzyme [11]. Generally, immobilized α-amylase is characterized by suitable hardness, density and porosity, which is more appropriate for practical applications [12]. α-Amylase has been covalently immobilized onto a wide variety of supports as poly(hydroxyethylmethacrylate), poly(methyl-methacrylate-acrylic acid) microspheres, zirconium membranes, epoxy group-containing porous membranes, phthaloyl chloride-containing amino group functionalized glass beads and a cyclic carbonate functional hybrid matrix [13,14,15,16,17,18]. Ion-exchange adsorption of α-amylase have also been performed on nitrocellulose membrane and chitosan beads [19,20].

The use of wool as immobilization support has been very limited [21,22]. 1,3,5-Triazine derivatives have found widespread applications in the pharmaceutical, textile, plastic and rubber industries [23,24]. The ease of displacement of chlorine atoms in cyanuric chloride by various nucleophiles groups (thiol, amino, imino and hydroxyl functions) results in stable linkages. The support material can have critical effect on the stability and the efficiency of enzyme immobilization. Recently, we published paper on the immobilization of horseradish peroxidase using wool as support which was activated by a multifunctional reactive center (cyanuric chloride) [25]. The activation of wool by cyanuric chloride permits a high multipoint covalent attachment. Therefore, this paper focused on immobilization of α-amylase from *T. harzianum* on wool activated by cyanuric chloride. Optimization and characterization of the immobilized enzyme compared with the soluble enzyme has been detected. *T. harzianum* α-amylase was previously purified and characterized with wide substrate specificity [26].

## 2. Results and Discussion

**Figure 1:**
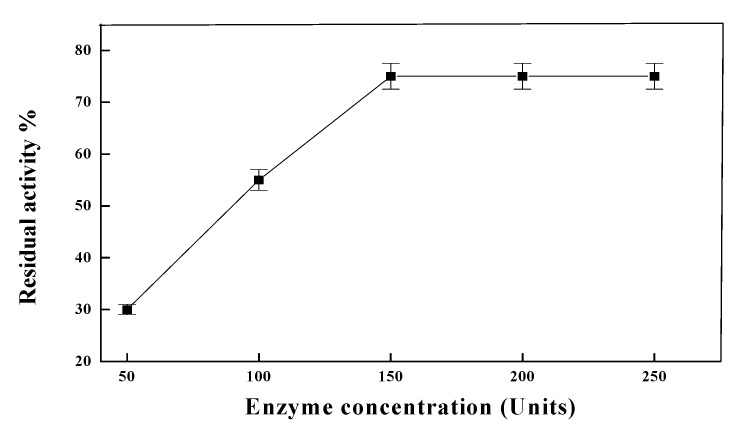
The effect of the soluble α-amylase concentration on the rate of immobilization. The immobilization process was performed at pH 7.2 with 4% (*w*/*v*) cyanuric chloride. Each point represents the mean of three experiments ± S.E. Please add one space between % and activity in the y axis in the Figure 1 and Figure 4.

The immobilization of *T. harzianum* α-amylase on wool activated by cyanuric chloride has been studied. In the present study, the effect of different concentrations of cyanuric chloride on the immobilization of *T. harzianum* α-amylase at pHs 5.5 or 7.2 was detected. An increase of cyanuric chloride concentration led to an increase in the immobilization efficiency. The maximum immobilization efficiency (70%) was detected at 4% cyanuric chloride and pH 7.2 (Table 1). Higher cyanuric chloride concentrations yielded a decrease in immobilization efficiency. The lowering in the retained activity by increasing the cyanuric chloride concentration could be attributed to the increase of multipoint attachments of the enzyme to the modified wool support which lead to changes in the structure of the enzyme. Although this could be produce an increase in enzyme stability it was discarded considering the decrease in activity. The effect of the soluble α-amylase concentration on the rate of immobilization was also studied. This study was performed under the optimum conditions of the immobilization process (pH 7.2 with 4% cyanuric chloride) mentioned above. Figure 1 show that the residual activity of immobilized enzyme increased with increasing the soluble α-amylase concentration, while the maximum rate of enzyme immobilization was detected at 150 units/g activated wool (75% residual activity). The same residual activity was detected at enzyme concentrations of 200 and 250 units.

**Table 1:** Effect of cyanuric chloride percentage and pH on the immobilization efficiency of *Trichoderma harzianum* α-amylase (200 units).

Cyanuric Chloride %	Immobilization Efficiency %	
	pH 5.5	pH 7.2
2	15 ± 0.7	25 ± 1.3
4	50 ± 2.8	70 ± 3.8
6	30 ± 1.7	45 ± 2.1
8	12 ± 0.7	22 ± 1.1

Each value represents the mean of three experiments ± S.E.

**Figure 2:**
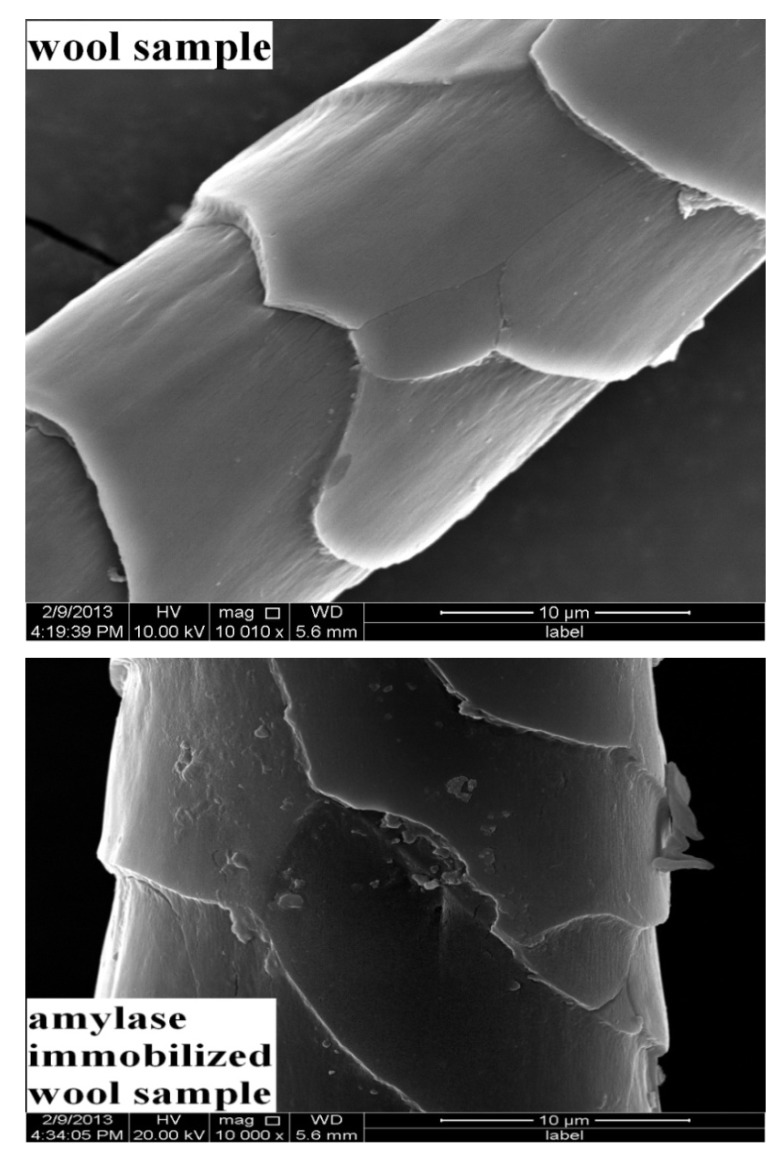
SEM images of wool sample (10,010×) and α-amylase immobilized wool sample (10,000×).

It is known that the morphology of wool is characterized by the presence of scales, which greatly contribute in protecting the wool from damage and affect other important properties of wool, such as luster and shrinkage. The SEM images in Figure 2 show that the scales on the untreated wool fiber (wool sample) are clear and arranged compactly around the fiber. The scales changed slightly after activation and α-amylase immobilization. The foreign materials observed in the SEM image could be attributed to the immobilized enzyme. The formation of new chemical bonds caused by binding of cyanuric chloride to wool and *T. harzianum* α-amylase has been confirmed by FTIR.

**Figure 3:**
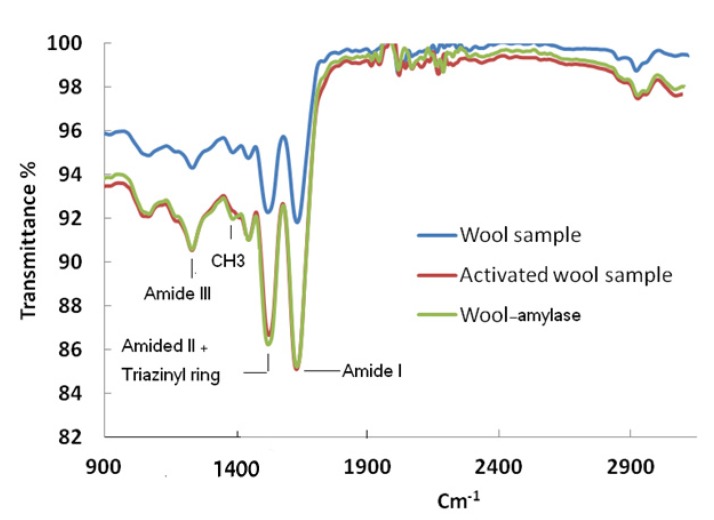
FT-IR spectra of the wool sample, the activated wool sample and the immobilized wool- α-amylase sample.

FTIR spectra of the wool sample, the activated wool sample and the immobilized wool- α-amylase sample are shown in Figure 3. Amide bands are observed at 1,630 cm^−1^ (C=O stretch, amide I), 1,520 cm^−1^ (N-H bend, amide II) and a weak band at 1,240 cm^−1^ (C-N stretch, amide III). These spectra showed a displacement and enlargement of the original band approximately 1,520 cm^−1^ related to the N-H deformation band, indicating the involvement of the amine groups in the chemical reaction. The characteristic peak of the triazinyl ring that appears at approximately 1,540 cm^−1^ confirms the success of activation and/or the enzyme immobilization reaction. The band at 1,390 cm^−1^ resulting from a CH_3_ symmetric bending mode appears with a slight increase in intensity with respect to the same band present in the activated wool sample, which adds evidence for the success of the immobilization of α-amylase on activated wool.

**Figure 4:**
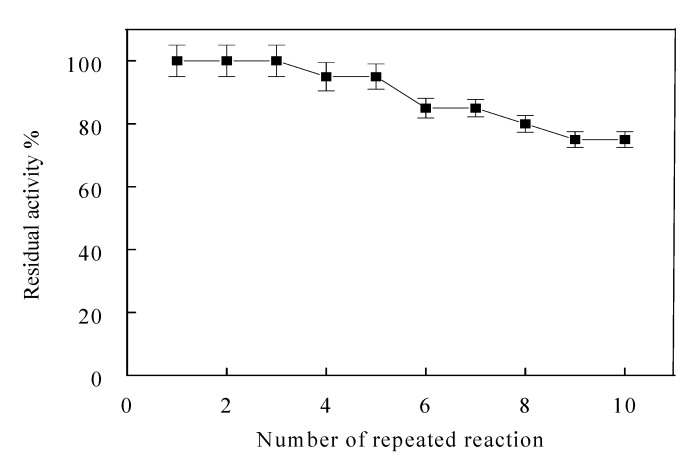
Reuse of wool-α-amylase. Each point represents the mean of three experiments ± S.E.

The most important advantage of immobilization is the possible repeated use of enzymes. Reusability of the immobilized α-amylase samples was examined by using the same conditions repeatedly 10 times and the measured activities are shown in Figure 4. It was observed that the immobilized enzyme demonstrated 75% activity after 10 runs. It was found that, the reuse capability of α-amylases was in the 50%–88% activity range after 5–25 runs [17,18,25,26,27].

**Figure 5:**
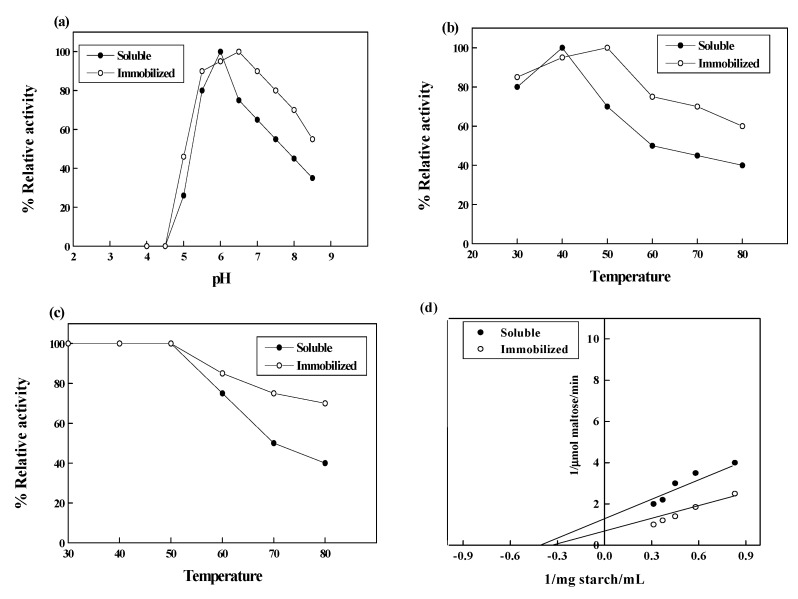
Optimum pH (**a**), optimum temperature (**b**), thermal stability (**c**) and Km (**d**) of soluble and immobilized α-amylases. Each point represents the average of two experiments.

The effect of pH on activity of soluble α-amylase and immobilized α-amylase was evaluated by incubating these preparations in the buffers of varying pH values ranged from 4.0 to 8.5 (Figure 5a). The soluble α-amylase and immobilized α-amylase exhibited maximum activity at pH values 6.0 and 6.5, respectively, with greater loss of activity recorded at pH 5.0 and pH 8.5 for soluble α-amylase (25% and 35% residual activity, respectively) compared with the immobilized enzyme (46% and 55% residual activity, respectively). It was deduced that the binding between wool and enzyme led to a conformation change of the microenvironment around the amylase, which was the main reason for the property changes of amylase after immobilization [18]. After immobilization, optimum pH of α-amylase was shifted from pH 6 to 8 [28,29,30] and pH 10 to 11 [31].

Figure 5b demonstrates the effect of temperature on the activity of soluble α-amylase and immobilized α-amylase. The optimum reaction temperatures for the soluble and immobilized α‑amylases were 40 °C and 50 °C, respectively. The immobilized α-amylase exhibited 60% of its activity at 80 °C, while the soluble enzyme retained 40% of its activity at the same temperature. The increase in optimum temperature was caused by the changing physico-chemical properties of the enzyme. After immobilization of α-amylase, a covalent bond was formed which may lead to a higher activation energy of the immobilized enzymes and an increase in substrate binding. One of the main reasons for enzyme immobilization is the anticipated increase in its stability toward various deactivating forces, due to restricted conformational mobility of the molecules following immobilization [15,32]. The increase in optimum temperature of α-amylases after immobilization has been reported in several studies [18,19]. The same temperature optimum (70 °C) was reported for free and immobilized α-amylase [30]. The thermal stability of the soluble and the immobilized α-amylases is shown in Figure 5c. The results showed that the soluble and immobilized α-amylase were thermally stable up to 50 °C, and retained 40% and 70% of the activity at 80 °C, respectively. Such an increase of thermal stability has been reported for a number of immobilized enzymes [19,30]. The thermal stability of enzymes might be drastically increased if they are attached to the complementary surface of a relatively rigid support in a multipoint way [33].

Various substrates, such as starch, glycogen, amylopectin, amylose, α-cyclodextrine and β-cyclodextrin were hydrolyzed by immobilized α-amylase (1 unit) with higher efficiencies than those of soluble α-amylase (1 unit) (Table 2), indicating that immobilization process didn’t affect the substrate binding site of the enzyme. Therefore, the cyanuric chloride didn’t cause any obstruction to substrate access at the binding site or at the active site. Variations in substrate specificities reflect differences in the affinities of the soluble and immobilized α-amylases. Similar results were observed by Pascoal *et al.* [34], where starches from various different sources were hydrolyzed by free and immobilized α-amylases from *Aspergillus niger*. Akkaya *et al.* [31] reported that free and immobilized α-amylases yielded the highest activity when corn starch was used as the substrate.

**Table 2:** The substrate specificity of soluble and immobilized α-amylases (1 unit).

Substrate	Relative Activity %	
	Soluble α-Amylase	Immobilized α-Amylase
Starch	95 ± 2.5	100 ± 3.1
Glycogen	80 ± 1.8	90 ± 2.6
Amylopectin	68 ± 2.1	80 ± 2.0
Amylose	70 ± 2.2	75 ± 1.5
α-Cyclodextrin	41 ± 1.6	53 ± 1.2
β-Cyclodextrin	42 ± 1.2	49 ± 1.6

The immobilized enzyme with starch is considered 100%. Each value represents the mean of three experiments ± S.E.

Generally, the affinity of a substrate toward an immobilized enzyme is lower than that of the free enzyme due to diffusional limitations, steric effects and ionic strength [32]. The ionic strength during immobilization may allow control of the penetration of the enzyme into the polymeric bed [35]. At high ionic strength, proteins can only become immobilized on areas of the protein which are able to simultaneously yield a very intense multipoint adsorption, even permitting the penetration in the polymeric bed structure, while using low ionic strength any area of the protein with negative charges may be involved in the adsorption. The change in the affinity of the enzyme for its substrate is also caused by structural changes in the enzyme introduced by the immobilization procedure and by lower accessibility of the substrate to the active site of the immobilized enzyme [13]. The affinity of substrate toward immobilized enzyme had been observed either higher or lower compared with soluble enzyme [15,18]. In the present study, compared with the soluble α-amylase, the affinity of immobilized α-amylase to starch decreased. Figure 5d shows that the Km values of the soluble α-amylase and the immobilized α-amylase were 2.5 mg starch/mL and 3.125 mg starch/mL, respectively.

The resistance of some enzymes against inactivation caused by metal ions can be considerably improved by immobilization [36,37]. The effect of metal ions on the activity of soluble and immobilized α-amylases was also studied (Table 3). Ca^2+^ and Co^2+^ enhanced the activity of soluble and immobilized α-amylases, whereas Ni^2+^ enhanced only the immobilized enzyme. The inhibition of the immobilized α-amylase by other metal ions was low, as compared to the soluble enzyme. Although Hg^2+^ caused strong inhibition for the activity of the soluble α-amylase, the immobilization of enzyme partially protected them from this harmful ion. This result is very important considering that inhibiting ions are often present in crude materials used in industrial processes. Similarly, Ca^2+^ enhanced the activity of free and immobilized α-amylase and Hg^2+^ inhibited both enzymes [33]. However, no effects were observed with immobilized α-amylase in the presence Ca^2+^, Cu^2+^, Fe^2+^ and Zn^2+^ compared with the inhibition of free enzyme [34].

**Table 3:** The effect of metal ions on the soluble and immobilized α-amylases (1 unit).

Metal Ion	Relative Activity %	
	Soluble α-Amylase	Immobilized α-Amylase
Control	100 ± 2.5	100 ± 3.1
Cu^2+^	70 ± 2.3	94 ± 2.8
Ni^2+^	78 ± 2.4	113 ± 2.5
Ca^2+^	109 ± 1.8	120 ± 2.3
Zn^2+^	91 ± 2.3	102 ± 3.2
Co^2+^	115 ± 1.7	125 ± 2.8
Pb^2+^	70 ± 2.2	90 ± 3.0
Hg^2+^	15 ± 0.6	60 ± 2.0

Each value represents the mean of three experiments ± S.E.

## 3. Experimental

### 3.1. T. harzianum α-Amylase

*T. harzianum* α-amylase was previously purified and characterized [26].

### 3.2. Preparation of Support

An ice-cooled solution of cyanuric chloride (2%–8% *w*/*v*) in acetone-water mixture (100 mL, 1:1) was prepared. Wool fabric (2 g) was added into this solution and left with shaking for 30 min at 0 °C. Then, sodium bicarbonate solution (10% *w*/*v*, 100 mL) was drop wisely added to the above reaction mixture while shaking within 30 min at 0 °C. The reaction mixture was further kept under shaking and at 0 °C overnight. The wool sample was removed from the shaker bath and washed several times with acetone, water and acetone, dried in ventilated hood and kept in a plastic bag in refrigerator ready for enzyme immobilization.

### 3.3. Immobilization Procedure

Enzyme immobilization was carried out by end over end rotation at 90 rpm on the activated wool using a solution of *Trichoderma harzianum* α-amylase (200 units) made in 50 mM sodium acetate buffer pH 5.5 or Tris-HCl pH 7.2 at room temperature during overnight. Aliquots of the supernatant were drawn up and the wool was dried at room temperature to verify the advancement of the immobilization. The relative activity % of immobilized enzyme was calculated from the following formula:

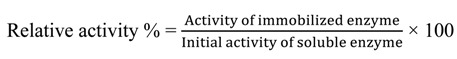
(1)


### 3.4. Characterization of Wool and Immobilized Wool-α-Amylase

Scanning electron microscopy (SEM) images of wool samples were examined with a Quanta FEG 450scanning electron microscope (FEI, Amsterdam, The Netherlands). The microscope was operated at an accelerating voltage of 10, 20 kV. The samples were placed on the double side carbon tape on Al- Stub and sputtered with a 20 nm thick gold layer (Jeol JFC-1600 Auto Fine Coater, Tokyo, city, Japan). The Attenuated Total Reflectance-Fourier Transform Infrared (ATR-FTIR) spectra for wool sample, activated wool sample and immobilized wool-α-amylase sample were recorded on a PerkinElmer spectrum 100 FT-IR spectrometer (New Jersey, NJ, US).

### 3.5. α-Amylase Assay

The α-amylase activity assay was carried out by DNS method [38], for both soluble and immobilized enzymes. One cm^2^ of wool was taken for routine assay of the activity of immobilized enzyme on wool. Wool was removed after 10 min incubation with 1 mL starch (1%) at 37 °C and 1 mL DNS was added for color development. The tube containing this reaction mixture was incubated in a boiling water bath for 10 min and then cooled in running tap water and the absorbance was recorded at 540 nm. One unit of activity was defined as the amount of enzyme required to produce 1 μmoL of maltose/min.

### 3.6. Reusability of Immobilized Enzyme

After each assay the immobilized enzyme preparation was taken out, washed with 50 mM sodium acetate buffer, pH 5.5 and stored overnight at 4 °C. The immobilized enzyme recovered by this procedure was used repeatedly. The activity determined for the first time was considered as control (100%) for the calculation of remaining percentage activity after each use.

### 3.7. Effect of pH and Temperature

The optimal pH and temperature for soluble α-amylase and immobilized α-amylase were made by using a pH ranged from 4.0 to 8.5 and a temperature range from 10 °C to 70 °C. The maximum activity was taken as 100% and % relative activity was plotted against different pH and temperature values. The thermal stability was investigated by measuring the activity of soluble α-amylase and immobilized α-amylase after 15 min of incubation at different temperatures prior to substrate addition. The % relative activity was plotted against different temperatures.

### 3.8. Determination of Kinetic Constant

The Km values were determined from Lineweaver-Burk plots by using different concentrations of starch as substrate (1.2–3.2 mg).

### 3.9. Substrate Specificity

Substrate specificity was investigated by incubating the soluble and immobilized α-amylases with 1% starch, glycogen, amylopectin, amylose, α-cyclodextrine and β-cyclodextrin.

### 3.10. Effect of Metal Ions

The effects of various metal ions (Cu^2+^, Ni^2+^, Ca^2+^, Zn^2+^, Co^2+^, Pb^2+^, Hg^2+^) on enzyme activity of soluble and immobilized α-amylases were determined by pre-incubating the enzyme with 2 mM metal ions for 15 min and then assaying the enzyme activity. The activity in absence of metal ions is taken as 100%.

## 4. Conclusions

The activated wool by cyanuric chloride as support causes less damage to the catalytic activity of α-amylase activity. The reuse capability of immobilized α-amylase was 75% of its activity after 10 runs. The wool-α-amylase exhibited a significant thermal stability and resistance toward metal ions compared with soluble enzyme. Various substrates were hydrolyzed by immobilized α-amylase with high efficiencies similar to those of soluble α-amylase indicating that immobilization didn’t affect the substrate binding site of the enzyme and could successfully be used in several applications especially the saccharification of starch.
